# Pre-Chemotherapy Dental Screening: Is There Additional Diagnostic Value for a Panoramic Radiograph?

**DOI:** 10.3390/dj11050122

**Published:** 2023-05-04

**Authors:** Judith A. E. M. Zecha, Alexa M. G. A. Laheij, Judith E. Raber-Durlacher, Anneke M. Westermann, Jan de Lange, Ludwig E. Smeele

**Affiliations:** 1 Department of Oral and Maxillofacial Surgery, Amsterdam UMC, University of Amsterdam, 1105 AZ Amsterdam, The Netherlands; 2 Department of Oral Medicine, Academic Centre for Dentistry Amsterdam, University of Amsterdam and VU University, 1081 LA Amsterdam, The Netherlands; 3 Department of Preventive Dentistry, Academic Centre for Dentistry Amsterdam, University of Amsterdam and VU University, 1081 LA Amsterdam, The Netherlands; 4 Department of Oncology, Amsterdam UMC, University of Amsterdam, 1105 AZ Amsterdam, The Netherlands; 5 Academic Centre for Dentistry Amsterdam, University of Amsterdam and VU University, 1081 LA Amsterdam, The Netherlands; 6 Department of Head & Neck Oncology & Surgery, Netherlands Cancer Institute-Antoni van Leeuwenhoek, 1066 CX Amsterdam, The Netherlands

**Keywords:** oral foci, dental focal infection, febrile neutropenia, dental screening, panoramic radiograph, cancer chemotherapy

## Abstract

Background: The oral cavity is a potential source of infectious complications in patients treated with myelosuppressive chemotherapy (CT). Pre-chemotherapy oral examination to identify foci of infection is recommended, but it is unclear whether this should include panoramic radiography. The present study aimed to evaluate the additional diagnostic merit of panoramic radiography as part of pre-CT oral screening. Methods: Patients with solid tumors scheduled to receive a myelosuppressive CT were eligible. The foci definition followed the guidelines of the Dutch Association of Maxillofacial Surgery. Oral foci assessed by clinical evaluation and panoramic radiography were compared. Results: In 33 out of 93 patients (35.5%), one or more foci were identified by clinical examination, whereas in 49.5% of patients, panoramic radiography showed pathology. In 19 patients, an oral focus was missed by clinical examination only, whereas in 11 patients, panoramic radiography indicated periodontal bone loss, but advanced periodontitis was not substantiated by clinical examination. Conclusions: Panoramic radiographs complement clinical examinations and have additional diagnostic value. Nevertheless, the additional merit seems small, and the clinical relevance may vary depending on the anticipated risk of developing oral complications and the need for detailed diagnosis and rigorous elimination of oral foci prior to the start of cancer therapy.

## 1. Introduction

Cancer patients treated with myelosuppressive chemotherapy (CT) may face serious complications due to their treatment. One potential side effect is febrile neutropenia (FN). FN is defined as fever that develops during the neutropenic phase [1], a phase in which the patient is immunocompromised and is more prone to infection. FN is associated with a high morbidity and mortality rate, particularly when caused by infection [2], and is therefore considered a medical emergency. As FN most commonly has an infectious cause, identifying a potential source of infection is a priority [3]. However, in only 20–30% of cases is a clinical infection documented [4]. Inflammatory and infectious oral conditions may induce or contribute to the development of FN. These, mostly chronic, oral infections can remain asymptomatic and present with only minimal signs and symptoms of inflammation during neutropenia, so they can be easily overlooked during inspection of the oral cavity when a patient presents with FN. Therefore, an oral examination to identify these oral foci prior to the start of myelosuppressive CT is recommended by many governmental and professional organizations involved in cancer care [5,6,7,8]. Potential foci of inflammation and infection include periodontitis, peri-implantitis, advanced dental caries with or without periapical involvement, pericoronitis, and root remnants [4]. However, there are no universally accepted protocols for the pre-chemotherapy diagnosis of oral foci [5,9].

To date, the literature is inconclusive about the benefits of radiographic imaging as part of oral screening using a panoramic radiograph. Epstein et al. [10] stated that panoramic radiography should be used only when indicated on the basis of a history of symptoms or clinical findings and not for screening purposes. In contrast, Gortzak et al. [11] concluded that panoramic radiographs are essential for dental screening in medically compromised patients, and Choi et al. [12] reported that a panoramic radiograph improved the efficacy of oral examination despite its insufficient accuracy for the diagnosis of dental caries, periodontal diseases, and other lesions. Recently, Yong et al. [13] suggested that dental screening should include panoramic radiograph and bitewing radiographs in patients treated with high-dose myeloablative chemotherapy regimens and receiving radiotherapy involving the dentition. In contrast, guidelines applying to dental examination in general dental practice recommend that panoramic radiographs should not be routinely made, as intraoral radiographs are considered the optimal radiographic examination [14,15]. However, the goals of taking radiographs for diagnostic purposes and treatment planning in general dental practice differ from those in cancer patients, including the identification of oral foci potentially causing FN, suggesting that different guidelines may apply.

Therefore, the aim of the present study was to determine the additional diagnostic merit of taking panoramic radiographs as part of dental screening prior to myelosuppressive CT.

## 2. Materials and Methods

This study was performed at the Department of Oral and Maxillofacial Surgery of the Amsterdam University Medical Center, location AMC. The Institutional Review Board approved this study (NL53440.018.15). All participants gave their written informed consent. The study was part of a prospective longitudinal observational study aimed at assessing the role of oral foci in FN development in patients with solid cancers and lymphoma treated with myelosuppressive CT that took place between December 2015 and December 2020. The results have been published elsewhere [16].

**Figure 1:**
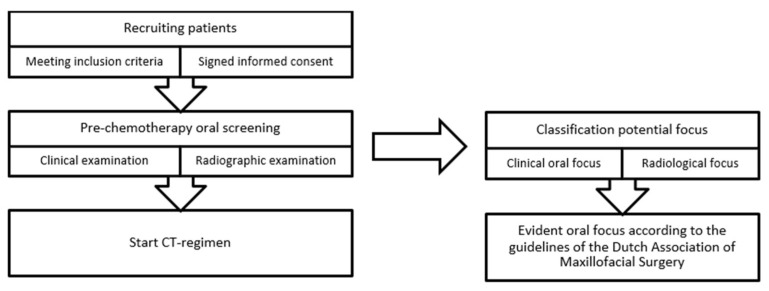
Flowchart study design. CT: chemotherapy.

Patients ≥ 18 years, with a (partial) natural dentition and/or dental implants scheduled to receive myelosuppressive CT for a solid tumor or lymphoma were eligible for inclusion. Patients with a history of radiotherapy in the head and neck region were excluded from participation. Screening consisted of a clinical examination of the oral mucosal tissues, the periodontium, and the dentition and evaluation of a digital panoramic radiograph (using Planmeca ProMax 2D 3), complemented by periapical radiographs when considered indicated. Clinical examinations and assessments of panoramic radiography were performed by one investigator (Judith Zecha DDS, MD; an experienced dentist about to complete an oral and maxillofacial surgery residency). Panoramic oral radiographs were viewed on screen. The clinical oral examinations and panoramic radiographs were evaluated separately for the presence and nature of oral and dental foci (see Figure 1). In order to avoid bias, these evaluations were performed independently of each other and took place at different times.

### 2.1. Pre-Chemotherapy Oral Screening

The clinical oral examination consisted of the following:

- Evaluation of dental habits (interval of regular dental visits, oral hygiene habits) and oral complaints over the last three months,
- Intraoral screening for mucosal and dental pathology (e.g., mucosal infections, caries/caries profunda, clinically visible root remnants, partially impacted teeth),
- Periodontal screening using the Dutch Periodontal Screening Index (DPSI), assessed per sextant [17]. The highest score was used for analysis,
- Screening for peri-implant mucositis and peri-implantitis.

On the panoramic radiograph, the following conditions were evaluated:

- Marginal alveolar bone loss,
- peri-implant alveolar bone loss,
- The presence of periapical lesions of endodontically and non-endodontically treated teeth,
- (Partially) impacted teeth,
- Root remnants,
- Other radiographic abnormalities.

In accordance with the guidelines of the Dutch Association of Maxillofacial Surgery [18], all pre-existing oral pathology that could contribute to the development of FN and infectious complications was noted as an oral focus (see Table 1). In this study, peri-implantitis was also considered to be a focus.

### 2.2. Statistical Analysis

Data were descriptive and the actual percentages of the parameters were calculated and described. Further statistical analysis was not performed, as this did not provide additional information.

## 3. Results

### 3.1. Patient Demographics

A total of 159 patients were eligible for inclusion. Of these, 93 agreed to participate. Reasons for not participating included: the full longitudinal study was too burdensome, already participating in several other clinical trials, already started with the CT regimen, and dental anxiety. Sixty-five patients (69.9%) were female, and the mean age was 54 (±15.6) years. Fifteen percent of the patients smoked. Most of the patients were diagnosed with a gynecological tumor (47.3%), followed by a tumor in the upper gastrointestinal tract (21.5%). More detailed demographic data are available in Appendix A. The majority of the patients brushed twice a day and visited the dentist every 6 months (see Appendix B).

### 3.2. Combined Findings of the Clinical Examination and the Panoramic Radiograph

All 93 patients underwent oral clinical and radiological evaluations. In none of these patients, additional periapical radiographs were considered necessary. The mean number of teeth was 25; seven patients had dental implants. Based on the combined findings of the clinical examination and the panoramic radiograph, 46 (49.5%) patients had a dental focus at the pre-treatment evaluation, of which 16 patients (17.2%) had more than one dental focus (see Table 2).

### 3.3. Findings of Clinical Evaluation Only

Most patients brushed their teeth twice daily and went to see the dentist on a regular basis. None of the patients had mucosal infections and no acute oral complaints were reported.

As shown in Table 2, one or more foci were identified in 33 patients (35.5%) by means of clinical examination only (e.g., without an additional radiograph). Advanced periodontitis (defined as the presence of one or more periodontal pockets ≥ 6 mm) was most commonly diagnosed; 14 of these 25 patients had furcation involvement. Peri-implantitis was diagnosed in one patient (1.1%). The majority of patients had a DPSI score of at most 2 (gingivitis and calculus) or 3 (periodontal pockets of 4–5 mm), not meeting the definition of a focus according to the Dutch Association of Maxillofacial Surgery.

### 3.4. Findings on the Panoramic Radiographs

In 49.5% of the patients, the panoramic radiograph showed one or more potential oral foci. In 29 patients (31.2%), one or more teeth with periapical pathology were identified (Table 2), but none of these teeth were reported to be symptomatic at the clinical evaluation. A total of 2361 teeth were evaluated, of which 63 teeth (2.67%) showed periapical pathology. Of the 2361 teeth, 135 teeth had been treated endodontically, of which 41 teeth (30.4%) showed periapical lesions (Table 3). In addition to periapical pathologies, panoramic radiographs revealed periodontal bone loss, impacted teeth, retained roots, profound caries and peri-implant related bone loss (Table 2). It should be noted, however, that radiographically identified periodontal bone loss was not considered to be a focus.

### 3.5. Added Diagnostic Value of Panoramic Radiography

In 19 patients, the panoramic radiograph revealed a potential focus (i.e., radiolucencies likely representing periapical pathology in the majority of cases) that was not detected by clinical evaluation. In 11 patients, periodontal bone loss was seen on the panoramic radiograph, but clinical evaluation did not reveal any pockets of ≥6 mm and thus did not meet the criteria of the Dutch Association of Maxillofacial Surgery to be considered as a dental focus. On the other hand, 11 out of the 25 patients clinically diagnosed with advanced periodontitis (pockets > 6 mm; DPSI 4) showed only mild to moderate alveolar bone loss on the panoramic radiograph.

## 4. Discussion

In this study, examining the added diagnostic value of panoramic radiographs in pre-chemotherapy oral screening, the identification of periapical pathology in the absence of clinical signs or symptoms was found to be the most prominent finding. In addition, panoramic radiographs provided extra information about the position of partially impacted teeth and retained roots, the extent of periodontal bone loss, and may be indicative of the presence of advanced caries lesions. Our results confirmed that panoramic radiography alone cannot replace clinical periodontal evaluation, although it has been suggested that it may play a role in screening patients toward a definitive periodontitis diagnosis [32].

Based on the combined findings of the clinical examination and the panoramic radiograph, 46 (49.5%) patients were diagnosed with a (potential) dental focus at the pre-treatment evaluation, of which 16 patients (17.2%) had more than one dental focus.

In 35.5% of patients, an oral focus was found with clinical examination alone, whereas in 49.5% of the patients, an oral focus (or potential oral focus) was identified by radiographic evaluation alone. In 19 patients diagnosed with an oral focus, the focus was identified only on the panoramic radiograph, suggesting that clinical examination alone would lead to underdiagnosis.

However, performing pre-treatment oral screening based on a panoramic radiograph alone is not advisable, as it may lead to inaccurate diagnoses, including over- and under-diagnosis. The majority of the foci identified on the panoramic radiograph were periapical lesions, suggesting periapical pathology. It should be noted that periapical pathologies of incisors may be underdiagnosed on panoramic radiographs [33]. Moreover, in our study, a focus was suspected in 11 patients based on periodontal bone loss on the panoramic radiograph, but clinical examination revealed no periodontal pockets ≥ 6 mm. This indicates that the radiological findings merely represented a history of periodontitis, or only mild to moderate periodontitis was present, not meeting the foci criteria of the Dutch Association of Maxillofacial Surgery. On the other hand, underdiagnosis may occur as 11 out of the 25 patients were clinically diagnosed with having pockets ≥ 6 mm, but only mild to moderate alveolar bone loss could be seen on the panoramic radiograph. Thus, a clinical examination is indispensable for the correct diagnosis of periodontitis, which is in line with the literature [34]. In the case of myelosuppressed cancer patients, this is particularly relevant because periodontal inflammation is a significant cause for the development of bacteremia [35] and FN [36]. In addition, clinical evaluation provides an opportunity to educate patients about the importance of maintaining good hygiene during and following cancer treatment.

The remaining root tips were radiographically identified in eight patients, two more than diagnosed by clinical examination. However, these radiographically detected root tips were covered by alveolar bone and, at clinical examination, found to be fully covered by oral mucosa without any signs of infection, which also points to the indispensable need for clinical interpretation.

Although comparisons of the prevalence of oral foci identified among different studies should be performed with great caution due to differences in focus definition, cancer diagnoses, differences between countries and socioeconomic factors, as well as the study size, the prevalence of oral foci found in our study falls within the wide range reported by Schuurhuis et al. [27] in a systematic review of head and neck cancer patients (20% to 79%). In addition, the prevalence of periodontitis reported in our study is in accordance with Nazir et al., with a reported prevalence ranging from 20 to 50% in patients with systemic diseases [37]. The percentage of patients with periapical pathology in our study was 31.2%, which is lower than the estimated prevalence of 52% of patients with periapical pathology globally [38]. As our study size was small, conclusions on the prevalence of oral foci cannot be drawn. Nevertheless, it can be noted that the accessibility and quality of dental care in the Netherlands is rather high [39]. Furthermore, bias may have occurred toward inclusion of dental-minded patients in our study.

Routinely taking panoramic radiographs seems to be the standard of care in oral screening prior to cancer therapy [25,26,40,41,42,43], despite the lack of supporting evidence. However, Epstein et al. [10] stated that panoramic radiography should not be conducted routinely for screening purposes. At first sight, our study seems to contradict this, as additional dental foci (i.g., peri-apical lesions) were identified with panoramic radiography. Nevertheless, the clinical significance of this finding remains to be assessed, as there is a growing body of evidence suggesting that asymptomatic periapical pathology is only rarely exacerbated in patients treated with myelosuppressive CT [16,25,30].

Clinical decision making and treatment of oral foci varies, depending on the type of oral focus, cancer diagnosis, the nature of cancer treatment, and the risk of acute and long-term oral complications [11,13,30]. Previous research showed that 25.2% of oncology patients undergoing dental assessment when presenting with FN required dental treatment due to dental abscesses and/or periodontitis [44]. This suggests that oral foci may play a role in causing FN and underscores the need for the dental evaluation of patients prior to the start of chemotherapy. However, the present study was part of a prospective observational study in which only one patient experienced an acute exacerbation of a chronic oral infection during the neutropenic phase, with 1.1% of the patients presenting with a dental focus prior to the start of the CT regimen [16]. The risk of developing oral infectious complications might be higher in patients treated with high-risk myelosuppressive CT or myeloablative CT regimens followed by stem cell transplantation (HCT) [41,42]. In general, these patients were screened by a dentist or maxillofacial surgeon prior to the start of treatment. Elad et al. [45] concluded after thorough analysis that dental treatment prior to the start of HCT prevents mortality in patients with hematologic malignancies. This points to the merit of including panoramic radiographs and, when indicated, combined with periapical radiographs when screening these patients [46].

However, the aggressiveness of dental therapy needed is increasingly subject of debate [16,25,40,47,48], as too rigorous treatment may unnecessarily mutilate the patient’s dentition and may put patients at risk for complications. Schuurhuis et al. [25] suggested that it is safe not to treat chronic oral infections in the absence of complaints in the previous three months before dental screening. More research is necessary before robust conclusions can be drawn about the need for detailed diagnosis, including panoramic radiography and rigorous elimination of foci in patients treated with different CT regimens and HCT regimens.

Patients diagnosed with malignancy in the head and neck region often receive radiotherapy (RT) as part of their treatment plan. Meticulous oral screening, including panoramic radiographs, enabling rigorous elimination of foci in this patient group is essential because of the long-term effects of RT, including the risk of developing rampant dental caries [49] and osteoradionecrosis of the jaw (ORN) [50].

Pre-treatment diagnosis and treatment of foci is also advised in cancer patients treated with intravenous bisphosphonates or other bone-modifying agents in the context of their primary cancer diagnosis (multiple myeloma) or metastatic disease (bone metastasis due to prostate or breast cancer). These patients are prone to develop medication-related osteonecrosis of the jaw (MRONJ), especially after (surgical) dental treatment [51]. Additionally, in these patient populations, the benefit of making panoramic radiographs to supplement clinical examination is evident.

To our knowledge, the current study is the first to solely evaluate the additional diagnostic value of the panoramic radiograph in pre-chemotherapy dental evaluation. Based on the results, a panoramic radiograph gives extra information about the periapical status of the dentition that cannot be detected with clinical examination alone when there are no symptoms (pain or sensitivity) or signs (fistulas, abscesses). Furthermore, a panoramic radiograph can give extra information about the presence of (partially) impacted teeth and retained roots, profound caries and the extent of periodontal bone loss or furcation involvement, but for the evaluation of the presence of periodontal disease, periodontal inflammation in particular, clinical examination is indispensable.

However, because of the small sample size, no robust conclusions can be drawn. Furthermore, periapical lesions were mostly found on the panoramic radiograph. However, intraoral radiographs are seen as the optimal radiographic examination method [14,15] and detecting periapical lesions in the incisor region is very difficult [33].

In conclusion, within the limitations of the small sample size, our study suggests that panoramic radiographs complement clinical screening for oral foci and have additional diagnostic value. Nevertheless, the additional benefit is small and routinely taking a panoramic radiograph in addition to the clinical oral examination seems not to have clinical relevance in patients scheduled to receive myelosuppressive CT for solid cancers.

Although not the subject of the present study, it should be noted that in patients receiving other types of cancer treatment (e.g., head and neck radiotherapy, CT with a high risk of myelosuppression or myeloablative conditioning for SCT, and/or bone-modifying agents), in which detailed diagnosis and more rigorous elimination of oral foci may be indicated, a panoramic radiograph should be considered in addition to clinical oral ex-amination. Future studies should be performed to validate our results and their clinical implications in larger as well as in other cancer patient populations.

## 5. Conclusions

Our study, within the limitations of the small sample size, suggests that panoramic radiographs complement clinical screening for oral foci and have additional diagnostic value. Nevertheless, the additional merit is small and routinely taking a panoramic radiograph seems not clinically relevant in patients scheduled to receive myelosuppressive CT for solid cancers.

## Figures and Tables

**Table 1:** Classification of oral focus [18] **.

advanced periodontitis (periodontal probing depth of ≥6 mm; DPSI 4)
profound dental caries
periapical pathology due to an infection of the root canal
(partially) impacted teeth
remaining roots with surrounding pathology

**: References used in the guideline [11,19,20,21,22,23,24,25,26,27,28,29,30,31]. DPSI: Dutch periodontal Screening Index.

**Table 2:** Clinical/radiological findings.

Findings of Combined Clinical and Radiological Evaluation (N = 93)					
			No. of patients (N)		Percentage (%)
Oral focus present		Yes	46		49.5
		No	47		50.5
Multiple oral foci		Yes	16		17.2
		No	77		82.8
Clinical focus (N = 93)			Radiological focus (N = 93)		
	No. of patients (N) *	Percentage (%)		No. of patients (N) *	Percentage (%)
Yes	33	35.5	Yes	46	49.5
Advanced periodontitis (pockets ≥ 6 mm)	25	26.9	Periodontal bone loss **	25	26.9
Furcation involvement	14	15.1	Periapical lesion	29	31.2
Partially impacted third molar	7	7.5	Partially impacted third molar	8	8.6
Retained roots	6	6.5	Retained roots	8	8.6
Profound caries	6	6.5	Profound caries	7	7.5
Peri-implantitis	1	1.1	Peri-implant bone loss	1	1.1
No	60	64.5	No	47	50.5
Dutch Periodontal Screening index [17]				No. of patients (N)	Percentage (%)
		Score 0		1	1.1
		Score 1		1	1.1
		Score 2		19	20.4
		Score 3−		39	41.9
		Score 3+		8	8.6
		Score 4		25	26.9
		Total		93	100

* Patients could have one or more different foci. ** Radiographic periodontal bone loss suggesting periodontitis or a history of periodontitis.

**Table 3:** Periapical pathology identified.

Periapical Pathology (N = 93)		
Number of teeth	2361	
Number of teeth with periapical lesions	63	
		2.67%
Number of endodontically treated teeth	135	
Number of endodontically treated teeth with periapical lesions	41	
		30.4%

(BMI = Body Mass Index, ASA = American Society of Anesthesiologists, WHO = World Health Organization, GI = Gastrointestinal).

## Data Availability

The data presented in this study are available on request from the corresponding author. The data are not publicly available due to privacy reasons. ClinicalTrials.gov Identifier: NCT02702583.

## Appendix A

**Patient demographics (N = 93)**			
		No. of patients (N)	Percentage (%)
Gender	Male	28	30.1
	Female	65	69.9
Age	Mean 54.0 years		
	Range 18–78 years		
	SD 15.6		
BMI	Mean 25.3		
	Range 16.8–44.3		
	SD 5.5		
Smoking	Yes	14	15.1
	No	58	62.4
	Quit	21	22.6
Current alcohol use	Yes	31	33.3
	No	62	66.7
ASA classification	ASA I	52	55.9
	ASA II	36	38.7
	ASA III	5	5.4
WHO performance status	WHO 0	56	60.2
	WHO 1	34	36.6
	WHO 2	3	3.2
Tumor and treatment characteristics (N = 93)			
		No. of patients (N)	Percentage (%)
Tumor subgroup	Gynecological	44	47.3
	Upper GI tract	20	21.5
	Sarcoma	12	12.9
	Urinary tract	6	6.5
	Lymphoma	5	5.4
	Breast	4	4.3
	Lower GI tract	2	2.2
Treatment goal	Curative	61	65.6
	Palliative	32	34.4
(BMI = Body Mass Index, ASA = American Society of Anesthesiologists, WHO = World Health Organization, GI = Gastrointestinal).			

## Appendix B

		**No. of Patients (N)**	**Percentage (%)**
Oral hygiene habits	Brushing; twice daily	70	75.3
	Brushing; daily	15	16.1
	Brushing; >twice daily	7	7.6
	Never	1	1.1
Dental visits	Twice a year	59	63.4
	Every year	22	23.7
	Sporadically	7	7.5
	Never	5	5.4
Patients with implants	Yes	7	7.5
	No	86	92.5
Number of teeth per patient	Mean 25		
	Range 8–32		
	SD 5.2		
